# Impact of hospital payment reform on rational use of antibiotics: a natural experiment

**DOI:** 10.3389/fpubh.2025.1638346

**Published:** 2025-09-01

**Authors:** Kun Zou, XiaoFan Peng, Yue Huang, JinMin Shi, ZhongCheng He, YiJia Hao, ChaoJie Liu, Yong Tang, ShaoYang Zhao, YongMu Jiang, Imti Choonara, LingLi Zhang

**Affiliations:** ^1^Department of Pharmacy, West China Second University Hospital, Sichuan University, Chengdu, Chengdu, Sichuan province, China; ^2^Evidence-Based Pharmacy Center, West China Second University Hospital, Sichuan University, Chengdu, Sichuan province, China; ^3^NMPA Key Laboratory for Technical Research on Drug Products In Vitro and In Vivo Correlation, Chengdu, Sichuan province, China; ^4^Key Laboratory of Birth Defects and Related Diseases of Women and Children, Ministry of Education, Chengdu, Sichuan province, China; ^5^Department of Epidemiology and Biostatistics, West China School of Public Health and West China Fourth Hospital, Sichuan University, Chengdu, Sichuan province, China; ^6^School of Business, Sichuan University, Chengdu, Sichuan province, China; ^7^Department of Mathematical Economics and Finance, Economics and Management School, Wuhan University, Wuhan, Hubei province, China; ^8^Guangyuan Human Resources and Social Security Bureau, Guangyuan, Sichuan Province, China; ^9^School of Public Health (Shenzhen), Shenzhen Campus of Sun Yat-sen University, Shenzhen, China; ^10^School of Psychology and Public Health, La Trobe University, Melbourne, VIC, Australia; ^11^School of Economics, Sichuan University, Chengdu, Sichuan province, China; ^12^School of Medicine, University of Nottingham, Nottingham, United Kingdom; ^13^Chinese Evidence-based Medicine Center, West China Hospital, Sichuan University, Chengdu, Sichuan province, China

**Keywords:** WHO AWaRe classification, rational use of antibiotics, hospital payment reform, natural experiment, DRG payment

## Abstract

**Introduction:**

While numerous studies have examined the effects of diagnosis-related groups (DRG) payment on hospital healthcare, evidence regarding its impact on the quality of hospital healthcare is poor and findings have been inconsistent. This study evaluates how DRG payment reform influences rational use of antibiotics in hospital using the World Health Organization (WHO) Access, Watch, Reserve (AWaRe) classification in China.

**Methods:**

We employed a natural experiment design with difference-in-differences analysis, comparing 10 hospitals implementing DRG payment in two pilot cities with 27 hospitals maintaining fee-for-service (FFS) payment in three control cities in Sichuan Province. Using medicine consumption data from 2016 to 2020 (185 hospital-years), we assessed total antibiotic use/expenditure, Access/Watch group proportions, and Access-to-Watch ratios.

**Results:**

Compared with FFS payment, DRG payment had no significant impact on the total quantity or expenditure of antibiotic use. However, DRG payment led to −13.01% (95% CI: −25.32% to - 0.70%) reduction of proportion of Access antibiotics use and 13.90% (95% CI: 1.97 to 25.83%) increase of proportion Watch antibiotics use. Subgroup analysis showed similar results in both secondary and tertiary hospitals, but greater in the former.

**Discussion:**

TDRG hospital payment reform may not affect the total quantity or expenditure of antibiotics use, but decrease the proportion of WHO Access antibiotics use and increase the Watch antibiotics use. Close monitoring and interventions are warranted to improve rational use of antibiotics for health systems during hospital payment reform.

## Introduction

The overuse and misuse of antibiotics has been a major public health challenge globally (1). The World Health Organization (WHO) listed antimicrobial resistance as one of 10 threats to global health (2). To improve monitoring and rational use of antibiotics globally, the WHO Expert Committee on Selection and Use of Essential Medicines developed the Access, Watch, Reserve (AWaRe) classification list of antibiotics in 2017 (3). By definition, Access antibiotics have low costs, clear efficacy, and a low likelihood of leading to drug resistance, making them highly cost-effective. It is recommended that antibiotics in the Access group should be available at all times as treatments for many types of common infections. With a broader spectrum and a higher resistance potential, Watch antibiotics are recommended only for specific, limited indications. Their use should be monitored for appropriateness as part of routine stewardship activities and reduced to avoid further development of resistance. The Reserve group consists of last-resort antibiotics for targeted use in multidrug-resistant infections when all alternatives have failed (3–7). The AWaRe classification provides useful tool and indicators to monitor and evaluate the rational use of antibiotics in terms of its volume and structure (6, 7).

Hospital payment reform, such as diagnosis-related groups (DRGs) payment, is being implemented or considered in China and many low- and middle- income countries (8). It has the potential to improve rational use of medicines by providing incentive for healthcare providers in removing unnecessary treatments or procedures in healthcare. Technically, it classified inpatient healthcare into several hundred to a thousand groups based on patients’ diagnoses, procedures, and risk factors (such as age), and predefines the reimbursement rate for each group. It allowed hospitals to retain surplus but also share cost of overspending. Theoretically, DRGs payment could motivate hospitals to improve providing more cost-effective interventions and reducing unnecessary diagnosis and treatment regimens and procedures, curbing overuse or misuse of antibiotics, for each patient compared with traditional fee-for-service (FFS) payment (9–12).

A large number of studies have been conducted to evaluated the impact of DRGs payment on hospital healthcare (13, 14). There is consistent evidence that DRGs payment has a mild effect in controlling medical expenditure, and a moderate effect in improving the efficiency of medical care by reducing hospital length of stay and increasing the number of patients being treated in defined period (15). However, evidence on the impact of DRGs payment on the quality of hospital healthcare is poor and findings have been inconsistent (16). A systematic review suggests that the DRG intervention had no demonstrable impact on patient quality of life and the quality of care received by patients may have decreased (14). For example, a study involving 297 US hospitals with over 14,000 patients found that the use of DRGs payment lead to reduction of in-hospital mortality and 30-day mortality (17). However, another study in Japan found that DRGs payment had no effect on the mortality rate of patients with acute myocardial infarction (AMI), but increased the readmission rate (18). A study in China found that the reform of DRGs payment in public hospitals was associated with significant reduction of using guideline recommend medicines (Aspirin and B-blocker), but had no effect on other quality indicators in patients with AMI compared to FFS payment. However, there has been no research on the impact of DRGs payment reform on rational use of antibiotics in hospital healthcare.

Therefore, we conducted this natural experiment with difference-in-differences (DID) analysis to evaluate the impact of DRGs payment reform on rational use of antibiotics in hospitals. We made two hypotheses: (1) compared with FFS payment, DRGs payment reform would reduce the total quantity and expenditure of antibiotic use in hospitals; (2) increase the proportion of Access antibiotics use (%), reduce the proportion of Watch antibiotics use (%) and increase the Access to Watch antibiotics ratio.

## Methods

### Study design

We employed a natural experiment design and DID analysis method to estimate the impact of the DRGs payment reform on antibiotics use in hospital healthcare compared to FFS payment system. The natural experiment with DID analysis commonly used to study the impact of health policy (19, 20).

In 2018, DRGs payment reform in hospital healthcare was piloted in Pan Zhi Hua city and Mei Shan city in Sichuan province. The DRG reform involved all inpatients who participate in basic medical insurance for urban workers, and that for urban and rural residents, which covered more than 95% of the population. The reform in Pan Zhi Hua city achieved full coverage of all hospitals in 2018. Mei Shan City started the DRGs payment reform from 2018, and achieved full coverage of all hospitals in 2020. Therefore, hospitals in Pan Zhi Hua city and Mei Shan city were selected as the experimental group. Hospitals in Neijiang, Leshan cities and Ganzi Tibetan Autonomous Prefecture, which continued the FFS payment system during the same period, were selected as the control group. The unit of observation in this study was hospital.

### Data source

Data were obtained from the Medicine Use Monitoring Database from Sichuan Health Commission, which contains basic information and medication use of hospitals in Sichuan province from 2016 to 2020. Secondary and tertiary hospitals in the above five cities were included and antibiotics usage data of all hospitals from 2016 to 2020 were extracted, including the volume and expenditure of antibiotics, the formulation unit and specification dosage of each category of antibiotics. The total volume of antibiotics, the volume of Access and Watch antibiotics are calculated by using defined daily dose (DDD) as the unit of measurement. After excluding missing values, 185 hospital-year data were finally obtained. In order to avoid the influence of extreme values on the research results, all the data in our study were winsorized with the level of 1%.

### Outcome measures

Eight outcomes derived from the WHO AWaRe classification were used to reflect the impact of DRGs payment on rational use of antibiotics, including: total volume and total expenditure of antibiotics, volume and expenditure of Access and Watch antibiotics (respectively), their shares in the total volume and total expenditure, and Access-to-Watch index as the ratio of the volume and expenditure between Access and Watch antibiotics (3). The total quantity of antibiotics, the quantity of Access and Watch antibiotics were calculated by using DDD as the unit of measurement.

### Covariables

Based on literature and availability of data (21), the following six control variables were finally introduced, including number of hospital beds per thousand people, number of doctors per thousand people, health expenditure per capita, degree of government support for local healthcare measured by the proportion of government health expenditure to total health expenditure, hospital grade (tertiary hospitals or secondary hospitals) and number of inpatients. Data of co-variables were obtained from Sichuan Statistical Yearbook and Sichuan Health Statistical Yearbook 2016–2020.

### Statistical analysis

We employed DID method to compare the pre- and post- changes between the experimental group (DRGs payment) and the control group (FFS payment), using the hospital as an observation unit. Multi-level linear regression analyses using fixed effect model were conducted using the following Equation 1:


(1) $$\begin{array}{l} Y_{i,t}=\alpha +\beta {\mathrm{DRG}}_{i,t}+\gamma \text{Control}\_{\mathrm{Var}}_{i,t}+\text{CityFE}+\\ \text{YearFE}+\text{HospitalFE}+\varepsilon_{i,t} \end{array}$$

Y*_i,t_* presents the dependent variables, among which total quantity and total expenditure are log transformed because the distributions of quantity and expenditure were skewed. The key explanatory variable is DRG_i,t_. It is a dummy variable indicating the DRG reform status. It equals one if the observations were from DRGs payment reform cities (Panzhihua city and Meishan city) and the time was after January 2018. Otherwise, it equals zero. The estimated coefficient β captures the average treatment effect of the DRGs payment reform. The Control_Var is the set of control variables, CityFE is the city fixed effect, YearFE is the year fixed effect, HospitalFE is the hospital fixed effect and ε is the disturbance. Subgroup analyses were conducted by level of hospitals (secondary and tertiary hospitals), to examine if the impact was different for the two level of hospitals. The parallel trends between the control group and the experimental group before implementation of the DRG reform were tested. We also conducted placebo test to guarantee the robustness of the regression results. Statistical analyses were two tailed, with a significance level of 5%. Standard errors were calculated based on maximum likelihood estimation. All analyses were conducted using Stata, version 17.0.

## Results

### Descriptive statistics

Table 1 shows the descriptive statistics of various variables, based on the observed values of experimental and control hospitals before (2016–2017) and after (2018–2020) the DRG payment reform at 2018 (see Appendix A, which shows the contrast before and after.)

**Table 1:** Summary of Antibiotics utilization in experimental and control hospitals before and after the introduction of DRGs Payment reform at 2018 (*N* = 185 hospital-year observations).

Characteristics	Pre-policy		Post-policy	
	Experimental	Control	Experimental	Control
Total hospital-year	20	54	30	81
Secondary hospital-year	8	34	12	51
Tertiary hospital-year	12	20	18	30
Total quantity of antibiotics, median (p25, p75), thousand DDD^a^	555.07 (311.21, 1479.26)	277.01 (93.57, 767.66)	473.80 (330.93, 1472.79)	292.33 (76.30, 816.05)
Total quantity of antibiotics (ln)^b^	13.37 (0.80)	12.54 (1.41)	13.34 (0.89)	12.50 (1.46)
Total expenditure of antibiotics, median (p25, p75), million yuan	9.32 (4.64, 22.87)	6.86 (1.25, 17.81)	8.95 (5.49, 32.03)	6.96 (0.97, 20.30)
Total expenditure of antibiotics (ln)^b^	15.41 (2.84)	15.65 (2.44)	15.74 (2.41)	15.67 (2.34)
Proportion of quantity of Access antibiotics, %	24.24 (19.23)	33.65 (21.30)	20.90 (15.47)	32.98 (20.83)
Proportion of expenditure of Access antibiotics, %	12.68 (6.75)	19.00 (15.45)	10.82 (59.3)	19.32 (12.70)
Proportion of quantity of Watch antibiotics, %	55.10 (18.88)	51.26 (18.32)	62.62 (15.98)	55.70 (18.77)
Proportion of expenditure of Watch antibiotics, %	62.56 (12.15)	58.55 (18.14)	65.84 (11.86)	62.86 (17.48)
Access to Watch quantity ratio	0.69 (1.0)	1.61 (3.81)	0.45 (0.62)	1.22 (2.87)
Access to Watch expenditure ratio	0.22 (0.12)	0.46 (0.64)	0.17 (0.11)	0.46 (0.64)

DRGs, diagnosis-related groups; DDD, defined daily dose.

a The total quantity of antibiotics, the quantity of Access and Watch antibiotics were calculated by using DDD as the unit of measurement.

b Since their distributions were skewed, the total quantity and total expenditure were ln transformed to be included in the regression model.

### Total quantity and total expenditure of antibiotics

In multivariate regression analysis model 1, DRG payment reform was significantly associated with increase of total quantity (mean difference [MD]: 1.04, 95%CI: 0.53 to 1.56%, *p* < 0.001), and total expenditure of antibiotics (MD: 1.09, 95%CI: 0.07 to 2.12%, *p* = 0.036), adjusted for hospital grade. However, multivariate regression analysis in model 2 adjusting for both hospital level and city level covariates, though DRG payment reform was still associated with increase of total quantity (MD: 0.32, 95%CI: −0.98 to 0.34%, *p* = 0.339) and total expenditure (MD: 0.63, 95%CI: −0.80 to 2.06%, *p* = 0.387) of antibiotics, the effect became non-significant (Table 2).

**Table 2:** Impact of hospital payment reform on hospital antibiotic utilization using multivariate difference-in-differences analysis.

Outcomes	Model 1^a^				Model 2^b^			
	β	95%	CI	*p*	β	95%	CI	*p*
Total quantity(ln)^c^	1.04	0.53	1.56	<0.001	0.32	−0.98	0.34	0.339
Total expenditure(ln)^c^	1.09	0.07	2.12	0.036	0.63	−0.80	2.06	0.387
Access quantity %	−14.59	−22.66	−6.53	<0.001	−13.01	−25.32	−0.70	0.038
Access expenditure %	−7.85	−13.08	−2.62	0.003	−1.41	−8.99	6.17	0.714
Watch quantity%	9.75	1.91	17.59	0.015	13.90	1.97	25.83	0.023
Watch expenditure %	4.11	−3.38	11.61	0.281	−1.87	−13.31	9.57	0.748
Access to Watch quantity ratio	−0.51	−1.82	0.78	0.434	−1.65	−3.60	0.30	0.098
Access to Watch expenditure ratio	−0.31	−0.55	−0.07	0.012	−0.02	−0.38	0.35	0.924

CI, confidence interval.

a In model 1, control variable is hospital grade.

b In model 2, control variables included hospital grade, number of hospital beds per thousand people, number of doctors per thousand people, Health expenditure per capita, and proportion of government financial support.

c Since their distributions were skewed, the total quantity and total expenditure were ln transformed to be included in the regression model.

### Proportion of access quantity and expenditure

In multivariate regression model 1, DRGs payment reform was associated with a significant decrease of Access quantity % (MD: −14.59, 95%CI: −22.66% to −6.53%, *p* < 0.001) and Access expenditure % (MD: −7.85, 95%CI: −13.08% to −2.62%, *p* = 0.003) compared with FFS payment. In multivariate regression model 2, the DRGs payment reform was still associated with significant decrease of Access quantity % (MD: −13.01, 95%CI: −25.32% to - 0.70%, *p* = 0.038) but non-significant decrease of Access expenditure % (MD: −1.41, 95%CI: −8.99 to 6.17%, *p* = 0.714; Table 2).

### Proportion of watch quantity and expenditure

In multivariate regression model 1, DRGs payment reform led to a significant increase of the proportion of Watch antibiotic quantity (MD: 9.75, 95%CI: 1.91 to 17.59%, *p* = 0.015) compared to FFS payment, with a non-significant increase of the proportion of Watch expenditure (MD: 4.11, 95%CI: −3.38 to 11.61%, *p* = 0.281). In multivariate regression model 2, DRGs payment reform was still significantly associated with increase of Watch antibiotics usage quantity (MD: 13.90, 95%CI: 1.97, to 25.83%, *p* = 0.023), and the increase of Watch antibiotics expenditure was still not significant (MD: −1.87, 95%CI: −13.31 to 9.57%, *p* = 0.748; Table 2).

### Access to watch index (ratio) of quantity and expenditure

In multivariate regression model 1, there was a non-significant decrease of Access to Watch quantity Ratio (MD: −0.51, 95%CI: −1.82 to 0.78, *p* = 0.434) but a significant decrease of Access to Watch expenditure ratio (MD: −0.31, 95%CI -0.55 to −0.07, *p* = 0.012) after the DRGs payment reform. In model 2, there was decrease of Access to Watch quantity ratio (MD: −1.65, 95%CI: −3.60 to 0.30, *p* = 0.098) and Access to Watch expenditure ratio (MD: −0.02, 95%CI: −0.38 to 0.35, *p* = 0.924) after the DRG payment reform, but the effects became non-significant for both outcomes (Table 2).

### Subgroup analysis

The results of the subgroup analysis by hospital grade showed that the impact of DRGs payment reform was consistent for both secondary and tertiary hospitals, and was similar to the results of the main analysis. However, it showed that the impact of DRGs payment reform was larger on secondary hospitals than tertiary hospitals (Table 3). For secondary hospitals, DRGs payment reform was associated with a significant increase of total quantity (MD: 1.76, 95% CI: 0.92% to 2.59, *p* < 0.001) and total expenditure (MD: 1.76, 95% CI: 0.92 to 2.59%, *p* < 0.001), but a significant reduction of Access quantity % (MD: −21.69, 95% CI: −34.63% to −6.74%, *p* = 0.004) and Access expenditure % (MD: −14.86, 95% CI: −24.13% to −5.59%, *p* = 0.002) and Access to Watch expenditure ratio (MD: −0.55, 95% CI:-0.95 to −0.15). In model 2, the impact of DRG payment was similar to that of model 1, it was still significantly associated with reduction of Access quantity % (MD: −22.96, 95% CI: - 45.07% to −00.84%, *p* = 0.042) and increase of Watch quantity % (MD: 21.38, 95% CI: 9.20 to 41.84%, *p* = 0.041), though the impact on other outcomes became non-significant. For tertiary hospitals, the impact of DRGs payment on outcomes of rational use of antibiotics was similar to that of secondary hospitals, but the effects were all non-significant (Table 3).

**Table 3:** Impact of hospital payment reform on hospital antibiotic utilization using multivariate difference-in-differences analysis by hospital grade.

Outcomes	Model 1^a^				Model 2^b^			
	β	95%	CI	*p*	β	95%	CI	*p*
Secondary hospital *n* = 105								
Total quantity (ln)^c^	1.76	0.92	2.59	<0.001	−0.71	−1.79	0.37	0.195
Total expenditure (ln)^c^	2.55	1.25	3.86	<0.001	0.59	−1.44	2.61	0.566
Access quantity %	−21.69	−34.63	−6.74	0.004	−22.96	−45.07	−00.84	0.042
Access expenditure %	−14.86	−24.13	−5.59	0.002	−1.16	−15.92	13.60	0.877
Watch quantity %	13.36	0.74	25.98	0.038	21.38	9.20	41.84	0.041
Watch expenditure %	7.03	−5.62	19.70	0.273	−7.01	−27.88	13.85	0.506
Access to Watch quantity ratio	1.05	−3.53	1.43	0.405	−4.17	−7.88	−0.45	0.028
Access to Watch expenditure ratio	−0.55	−0.95	−0.15	0.008	0.04	−0.61	0.68	0.908
Tertiary hospital *n* = 80								
Total quantity (ln) ^c^	0.05	−0.48	0.58	0.848	0.20	−0.94	0.54	0.597
Total expenditure (ln) ^c^	1.36	−2.87	0.15	0.078	0.02	−1.96	1.99	0.988
Access quantity %	−4.46	−10.93	1.99	0.172	−5.74	−14.51	3.02	0.196
Access expenditure %	−0.71	−4.07	2.63	0.670	−2.91	−7.32	1.50	0.193
Watch quantity %	3.02	−6.09	12.13	0.511	8.69	−4.21	21.60	0.184
Watch expenditure %	2.26	−5.94	10.46	0.584	3.64	−7.70	14.99	0.524
Access to Watch quantity ratio	−0.17	−0.38	0.05	0.123	−0.20	−0.49	0.09	0.180
Access to Watch expenditure ratio	−0.05	−0.31	0.20	0.683	−0.12	−0.49	0.25	0.521

CI, confidence interval.

a In model 1, no adjustment of other variables.

b In model 2, control variables included hospital grade, number of hospital beds per thousand people, number of doctors per thousand people, Health expenditure per capita, and proportion of government financial support.

c Since their distributions were skewed, the total quantity and total expenditure were ln transformed to be included in the regression model.

### Parallel trend test

The results indicate that the coefficients of the relative time dummy variables before the policy change are not significant. This suggests that before the policy piloted, there were no significant differences between the experimental and control groups in terms of the total expenditure and quantity of antibiotic usage, the proportion of Access and Watch usage relative to the total quantity, and the proportion of Access and Watch expenditures relative to the total expenditure, indicating the parallel trend assumption was satisfied (Appendix B).

### Placebo test: random generation of experimental group

Taking the results of the dependent variable Watch quantity % an example, the *β* random generated during the randomization process is mainly concentrated around 0, whereas the estimated coefficient for the actual policy is 0.13, showing a significant difference from the placebo test results. This also suggests that the quantitative assessment results in this study are not significantly influenced by this potential factor, indicating results were robust (Appendix C).

## Discussion

There were three main findings. First, DRGs payment system had no significant impact on the total quantity and total expenditure of antibiotic usage in hospitals compared to FFS payment. Second, DRGs payment reform resulted in a significant reduction in the proportion of quantity of Access antibiotics, and an increase in the proportion of quantity of Watch antibiotic. Third, the impact of the DRGs payment reform might be greater in secondary hospitals than in tertiary hospitals.

This study found that DRGs payment reform did not affect the total quantity or expenditure of antibiotics used in hospitals. In theory, DRGs payment can offer incentive of cost-containing by removing over-treatment compared with FFS payment, which may reduce the quantity and cost of antibiotic use (22). However, our study found that DRGs payment did not lead to a significant decrease in total quantity and cost of antibiotic utilization compared to FFS payment. These findings were consistent with findings from study of Dong and colleagues, which found that DRGs payment reform did not affect use of antibiotics (23). One reason may be that in the DRG payment reform, the standard of payment for each DRG was calculated according to the retrospective data of medical costs of former inpatients of reform hospitals, and allowed a corresponding percentage growth rate every year, which to a certain extent considered the historical continuity of the expenses of medical services (24). Therefore, the pressures on hospitals to reduce cost may not be so great, and may not have a dramatic impact on provision of medical services and antibiotics, especially in the short-term.

Another important finding of our study is that the DRGs payment reform resulted in a decrease of the percentage of Access antibiotic usage and an increase of the percentage of Watch antibiotic usage, which implied that the pattern of rational use of antibiotics was worse (25). According to the WHO AWaRe classification of antibiotics, Access antibiotics referred to the antibiotics that should be widely available, cheaper, therapeutically effective, narrow-spectrum with a lower risk of inducing resistance, and should be used as the preferred first-line drugs. On the other hand, Watch antibiotics are broader-spectrum antibiotics with a wider antimicrobial spectrum, and have a higher likelihood of resistance. Therefore, WHO has endorsed the priority use of Access antibiotics, and set a goal of 60% of its share in total use of antibiotics (15). The significant decrease of share of Access antibiotics after transition from FFS payment to DRGs payment is worrisome.

There may be several reasons for the above findings. First, the antibiotic classification management lists in China are different from the WHO AWaRe classification (26). As a management tool to deal with the challenge of bacterial resistance in various regions and countries, the AWaRe classification focuses on the situation of bacterial resistance (27, 28) and considers the effectiveness and accessibility of the included drugs (29). In the past decade, China has established a comprehensive management system for antimicrobial stewardship. The provincial health administrations were authorized to develop a hierarchical management catalog based on a national catalog, considering the characteristics, efficacy, safety, bacterial resistance, and drugs price of various antibiotics, among other factors. In the national catalog, antibiotic preparations were classified into non-restricted, restricted and highly-restricted classifications (30). Prior authorization is not needed to use non-restricted antibiotics, which physicians can apply directly based on clinical needs. However, prescriptions of restricted antibiotics must be authorized by the attending (or chief) physician before use, while highly-restricted antibiotics can be used only when the evidence and indications are clear, and prior authorization is needed by at least two authorized senior physicians.

In total, there are 182 kinds of antibiotics included in the national catalogue of antibiotics and the AWaRe classification catalogue. However, 91 antibiotics listed in the national catalogue were not in the AWaRe classification catalogue, and 93 antibiotics listed in the AWaRe classification catalogue were not included in the Chinese national catalogue. In terms of categories, 30 of the 90 kinds of antibiotics listed as non-restricted class in the Chinese national catalogue were classified as within the Watch group and 1 within the Reserve group in the WHO AWaRe classification. A total of 98 antibiotics were classified to highly-restricted antibiotics in the Chinese national catalogue, of which 9 were classified as Access antibiotics in the WHO AWaRe classification (26). The large difference between the two catalogs may be one of the reasons for our findings (31). However, we have supplemented our Appendix D with information on the impact of DRG under the Chinese antibiotic classification, which shows a significant downward trend in the non-restricted to restricted quantity ratio, consistent with changes in antibiotic use under the AWaRe classification.

Secondly, under the DRGs payment, physicians may tend to use Watch antibiotics for the sake of time and short-term clinical benefit. There was strong evidence that DRGs payment created an incentive for hospitals to improve clinical efficiency, to reduce length of stay and increase the number of admissions, which may be transferred to physicians (22, 32). Because of their broad spectrum, Watch antibiotics are more likely to be empirically used by physicians, to secure a faster and more efficient cure and shorter hospital stay, especially when results of etiology examination were not feasible or yet obtained. The possible harms are worthy of attention.

These findings imply that more measures are needed to improve the rational use of antibiotics. DRGs payment may not be the best way to facilitate rational prescribing.

The incentive function of the payment system may be fully utilized to promote healthcare providers to improve rational use of antibiotics. The experience from many countries to use pay-for-performance with indicators and incentives for rational use of antibiotics may be considered and adopted (15, 33–35). Second, the regulation of antibiotics may be improved. The hierarchical antibiotics management list may be refined in line with the WHO AWaRe classification, informed by evidence-based medicine and account to population needs, prevalence of drug resistance in China (36). This will not only be in line with international best practice, but also be better applied to China’s antibacterial drugs management system.

At the same time, the health authorities, as the administrator and regulator of the rational use of antibiotics, may take additional economic or behavioral interventions to improve the use of antibiotics. For example, public reporting of health care performance can positively influence antibiotic prescribing patterns of physicians, and thus may promote the rational use of antibiotics (37, 38). Besides, the theory of behavioral economy could be applied with hospital information system (HIS) to reduce unnecessary use of antibiotics. For example, a previous study found that behavioral economic intervention, which required doctors to specify the detailed reasons in the electronic HIS, when prescribing antibiotic after a ceiling of antibiotics prescribed every day, significantly reduced the quantity of antibiotics prescriptions (39).

Third, clinical pharmacists may contribute more in promoting rational use of antibiotics. By increasing the responsibility and function of hospital drug committees, pharmacy departments and clinical pharmacists may make a greater contribution in rational prescribing, using monitoring, prescription audit, prior authorizing (of restricted antibiotics), regular training, education, consultation for clinicians and patients on rational use of antibiotics, which have shown to be effective measures (40).

During the period of 2016–2020, China has also introduced some policies that control medical expenses, such as zero-mark up policy implemented in 2017 in tertiary hospitals, the two-invoice system policy and the pooling procurement policy implemented since 2019 (41, 42). Of course, the COVID-19 pandemic that began at the end of 2019 may also have a significant impact on the use of antibiotics (43). Assuming that these events have almost the same effect on the use of antibiotics in different public hospitals, in this study, we first divide the experimental group and the control group according to whether they started the DRGs payment reform, and applying DID analysis to examine the rational use of antibiotics, which may control these confounding policies to some extent.

This study had several limitations. First, due to the availability of data, only hospitals with complete data from 2016 to 2020 in the cities were chosen (the percentage of complete data is 96.98%). Therefore, selection bias may not be ruled out. Second, this study only evaluated the short-term impact of DRGs payment reform on rational use of antibiotics in hospitals. Its long-term impact warrant further investigation. Third, this study revealed the impact of the DRGs payment reform on rational use of antibiotics in hospitals, but the underlying mechanisms of these behavioral changes is of interest and warrant in-depth research to inform tailored interventions.

## Conclusion

DRGs payment reform may not affect the total quantity or expenditure of antibiotics use compared with FFS payment. However, it may negatively affect the structure of antibiotics utilization by reducing the proportion of Access antibiotics and increasing the proportion of Watch antibiotics, which are more likely to introduce bacteria-resistance. In transition from FFS payment to DRGs payment, more measures including regulation improvement, economic, behavioral, and pharmacist interventions might be warranted to promote the rational use of antibiotics in hospitals.

## Data Availability

The raw data supporting the conclusions of this article will be made available by the authors, without undue reservation.

## References

[1] Laxminarayan R Duse A Wattal C Zaidi AKM Wertheim HFL Sumpradit N. Antibiotic resistance-the need for global solutions. Lancet Infect Dis. (2013) 13:1057–98. doi: 10.1016/S1473-3099(13)70318-9, PMID: 24252483

[2] World Health Organization. Ten threats to global health in 2019. (2019). Available at: https://www.who.intvietnam/news/feature-stories/detail/ten-threats-to-global-health-in-2019. (Accessed 28 Jul 2023).

[3] Hsia Y Lee BR Versporten A Yang Y Bielicki J Jackson C. Use of the WHO access, watch, and reserve classification to define patterns of hospital antibiotic use (AWaRe): an analysis of paediatric survey data from 56 countries. Lancet Glob Health. (2019) 7:e861–71. doi: 10.1016/S2214-109X(19)30071-3, PMID: 31200888

[4] Word Health Organization. Adopt AWaRe: handle antibiotics with care. (2015). Available at: https://adoptaware.org/#resources (Accessed 28 Jul 2023).

[5] Adekoya I Maraj D Steiner L Yaphe H Moja L Magrini N. Comparison of antibiotics included in national essential medicines lists of 138 countries using the WHO access, watch, reserve (AWaRe) classification: a cross-sectional study. Lancet Infect Dis. (2021) 21:1429–40. doi: 10.1016/S1473-3099(20)30854-9, PMID: 34332706

[6] Sharland M Gandra S Huttner B Moja L Pulcini C Zeng M. Encouraging AWaRe-ness and discouraging inappropriate antibiotic use—the new 2019 essential medicines list becomes a global antibiotic stewardship tool. Lancet Infect Dis. (2019) 19:1278–80. doi: 10.1016/S1473-3099(19)30532-8, PMID: 31782385

[7] Alshareef H Alanazi A Alatawi N Eleshmawy N Ali M. Assessment of antibiotic prescribing patterns at dental and primary health care clinics according to WHO access, watch, reserve (AWaRe) classification. Am J Infect Control. (2023) 51:289–94. doi: 10.1016/j.ajic.2022.07.009, PMID: 35870657

[8] Mehmood A Ahmed Z Ghailan K Dohare S Varghese J Azeez FK. Implementation of healthcare financing based on diagnosis-related group in three WHO regions; western Pacific, south East Asia and eastern Mediterranean: a systematic review. J Health Manag. (2023) 25:404–13. doi: 10.1177/09720634231168250

[9] Chang W Yan X Ling H Liu T Luo A. A study of the types and manifestations of physicians' unintended behaviors in the DRG payment system. Front Public Health. (2023) 11:1141981. doi: 10.3389/fpubh.2023.1141981, PMID: 37441652 PMC10333571

[10] Jian W Lu M Chan KY Poon AN Han W Hu M. Payment reform pilot in Beijing hospitals reduced expenditures and out-of-pocket payments per admission. Health Aff. (2015) 34:1745–52. doi: 10.1377/hlthaff.2015.0074, PMID: 26438752

[11] Goldfield N. The evolution of diagnosis-related groups (DRGs): from its beginnings in case-mix and resource use theory, to its implementation for payment and now for its current utilization for quality within and outside the hospital. Qual Manag Health Care. (2010) 19:3–16. doi: 10.1097/QMH.0b013e3181ccbcc3, PMID: 20042929

[12] National Health Commission of the People’s Republic of China. Text record of the media communication meeting on clinical pathway Management of the National Health Commission of the people’s republic of China. (2019). Available at: http://www.nhc.gov.cn/xcs/s3574/201708/a3ea7d5f6efa4a44bf338f1d55e60749.shtml. (Accessed 6 Sep 2023).

[13] Milcent C. From downcoding to upcoding: DRG based payment in hospitals. Int J Health Econ Manag. (2021) 21:1–26. doi: 10.1007/s10754-020-09287-x, PMID: 33128657

[14] Pott C Stargardt T Frey S. Does prospective payment influence quality of care? A systematic review of the literature. Soc Sci Med. (2023) 323:115812. doi: 10.1016/j.socscimed.2023.115812, PMID: 36913795

[15] Chen Y Zhang X Yan J Qian M-c Ying X-h. Impact of diagnosis-related groups on inpatient quality of health care: a systematic review and meta-analysis. Inquiry-the J Health Care Organization Provision and Financing. (2023) 60:469580231167011. doi: 10.1177/00469580231167011 PMC10126696 37083281

[16] Jian W Lu M Liu G Chan KY Poon AN. Beijing's diagnosis-related group payment reform pilot: impact on quality of acute myocardial infarction care. Soc Sci Med. (2019) 243:112590. doi: 10.1016/j.socscimed.2019.112590, PMID: 31683116

[17] Kahn KL Keeler EB Sherwood MJ. Comparing outcomes of care before and after implementation of the DRG-based prospective payment system. Jama-J American Medical Association. (1990) 264:1984–8. 2120477

[18] Hamada H Sekimoto M Imanaka Y. Effects of the per diem prospective payment system with DRG-like grouping system (DPC/PDPS) on resource usage and healthcare quality in Japan. Health Policy. (2012) 107:194–201. doi: 10.1016/j.healthpol.2012.01.002, PMID: 22277879

[19] Anderson M Molloy A Maynou L. Evaluation of the NHS England evidence-based interventions programme: a difference-in-difference analysis. BMJ Qual Saf. (2023) 32:90–9. doi: 10.1136/bmjqs-2021-014478, PMID: 35393354 PMC9887378

[20] Yip W Eggleston K. Provider payment reform in China: the case of hospital reimbursement in Hainan province. Health Econ. (2001) 10:325–39. doi: 10.1002/hec.602, PMID: 11400255

[21] Zang X Zhang M Wei S. Impact of public hospital pricing reform on medical expenditure structure in Jiangsu, China: a synthetic control analysis. BMC Health Serv Res. (2019) 19:512. doi: 10.1186/s12913-019-4357-x, PMID: 31337396 PMC6651988

[22] Zou K Li H-Y Zhou D Liao Z-J. The effects of diagnosis-related groups payment on hospital healthcare in China: a systematic review. BMC Health Serv Res. (2020) 20:112. doi: 10.1186/s12913-020-4957-5, PMID: 32050962 PMC7017558

[23] Dong Q. The influence of DRG implementation on hospitalization expenses in tertiary hospitals [doctor]. Beijing: Beijing University of Chinese Medicine (2021).

[24] Congress US. Medicare's prospective payment system: strategies for evaluating cost, quality, and medical technology. (1996). Available at: http://www.theblackvault.com/documents/ota/Ota_4/DATA/1985/8516.PDF (Accessed 1 Nov 2023).

[25] Hsia Y Sharland M Jackson C Wong ICK Magrini N Bielicki JA. Consumption of oral antibiotic formulations for young children according to the WHO access, watch, reserve (AWaRe) antibiotic groups: an analysis of sales data from 70 middle-income and high-income countries. Lancet Infect Dis. (2019) 19:67–75. doi: 10.1016/S1473-3099(18)30547-4, PMID: 30522834

[26] Yang Y Ling K Zhang X Du K Zhang W. Comparative analysis of domestic classification management list for clinical use of antibiotics versus WHO AWaRe classification list of antibiotics. China Pharm. (2022) 33:2945–51. doi: 10.6039/j.issn.1001-0408.2022.24.01

[27] World Health Organization. WHO releases the 2019 AWaRe classification antibiotics. (2019). Available at: https://www.who.int/news/item/01-10-2019-who-releases-the-2019-aware-classification-antibiotics. (Accessed 26 Dec 2023).

[28] World Health Organization. WHO antibiotic categorization. (2023). Available at: https://aware.essentialmeds.org/groups (Accessed 25 Dec 2023).

[29] World Health Organization. The selection and use of essential medicines. (2019). Available at: https://apps.who.int/iris/rest/bitstreams/1091561/retrieve. (Accessed 25 Dec 2023).

[30] National Health Commission of the People's Republic of China. Administrative Measures for the Clinical Use of Antibacterial Drugs. (2012). Available at: https://www.gov.cn/gongbao/content/2012/content_2201890.htm (Accessed 28 Jul 2023).

[31] Wushouer H Zhou Y Zhang W. Inpatient antibacterial use trends and patterns, China, 2013-2021. Bull World Health Organ. (2023) 101:248–261B. doi: 10.2471/BLT.22.288862 37008266 PMC10042087

[32] Agarwal R Liao JM Gupta A Navathe AS. The impact of bundled payment on health care spending, utilization, and quality: a systematic review. Health Aff (Millwood). (2020) 39:50–7. doi: 10.1377/hlthaff.2019.00784, PMID: 31905061

[33] Busse R Geissler A Aaviksoo A. Diagnosis related groups in Europe: moving towards transparency, efficiency, and quality in hospitals? BMJ Brit Med J. (2013) 346:19–22. doi: 10.1136/bmj.f3197 23747967

[34] Department of Health. Using the Commissioning for Quality and Innovation (CQUIN) Payment Framework. (2012). Available at: https://www.gov.uk/government/publications/using-the-commissioning-for-quality-and-innovation-cquin-payment-framework-guidance-on-new-national-goals-for-2012-13 (Accessed 25 Dec 2023).

[35] Feng Y Kristensen SR Lorgelly P Meacock R Sanchez MR Siciliani L. Pay for performance for specialised care in England: strengths and weaknesses. Health Policy. (2019) 123:1036–41. doi: 10.1016/j.healthpol.2019.07.007, PMID: 31405615

[36] Budd E Cramp E Sharland M Hand K Howard P Wilson P. Adaptation of the WHO essential medicines list for national antibiotic stewardship policy in England: being AWaRe. J Antimicrob Chemother. (2019) 74:3384–9. doi: 10.1093/jac/dkz321, PMID: 31361000

[37] Liu C Zhang X Wan J. Public reporting influences antibiotic and injection prescription in primary care: a segmented regression analysis. J Eval Clin Pract. (2015) 21:597–603. doi: 10.1111/jep.12343, PMID: 25902726

[38] Tang Y Liu C Zhang X. Performance associated effect variations of public reporting in promoting antibiotic prescribing practice: a cluster randomized-controlled trial in primary healthcare settings. Prim Health Care Res Dev. (2017) 18:482–91. doi: 10.1017/S1463423617000329, PMID: 28606190

[39] Richards AR Linder JA. Behavioral economics and ambulatory antibiotic stewardship: a narrative review. Clin Ther. (2021) 43:1654–67. doi: 10.1016/j.clinthera.2021.08.004, PMID: 34702589 PMC8612959

[40] Lambert M Smit CCH De Vos S Benko R Llor CP John W. A systematic literature review and meta-analysis of community pharmacist-led interventions to optimise the use of antibiotics. Br J Clin Pharmacol. (2022) 88:2617–41. doi: 10.1111/bcp.15254 35112381 PMC9313811

[41] Mao W Huang Y Chen W. An analysis on rational use and affordability of medicine after the implementation of National Essential Medicines Policy and zero mark-up policy in Hangzhou, China. PLoS One. (2019) 14:e0213638. doi: 10.1371/journal.pone.0213638, PMID: 30870490 PMC6417690

[42] Yin J Wu C Wei X Sun Q. Antibiotic expenditure by public healthcare institutions in Shandong Province in China, 2012-2016. Front Pharmacol. (2018) 9:1396. doi: 10.3389/fphar.2018.01396, PMID: 30559665 PMC6287473

[43] Hu Y Wei X Zhu Q Li L Liao C Jiang G. COVID-19 pandemic impacts on humans taking antibiotics in China. Environ Sci Technol. (2022) 56:8338–49. doi: 10.1021/acs.est.1c07655, PMID: 35675530

