# Supplementary material for: Impact of hospital payment reform on rational use of antibiotics: a natural experiment
Source: Front Public Health. 2025 Sep 1;13:1638346. doi: 10.3389/fpubh.2025.1638346 (PMC12433986; doi:10.3389/fpubh.2025.1638346)
Supplement: Supplementary file 1 [file Data_Sheet_1.docx]

**Appendices**

Appendix A

Fig. A.1 Use of antibiotics before and after hospital DRGs payment reform

Appendix B: Parallel Trend Test

Fig. B.1 Results of $QTY\_Access$ %


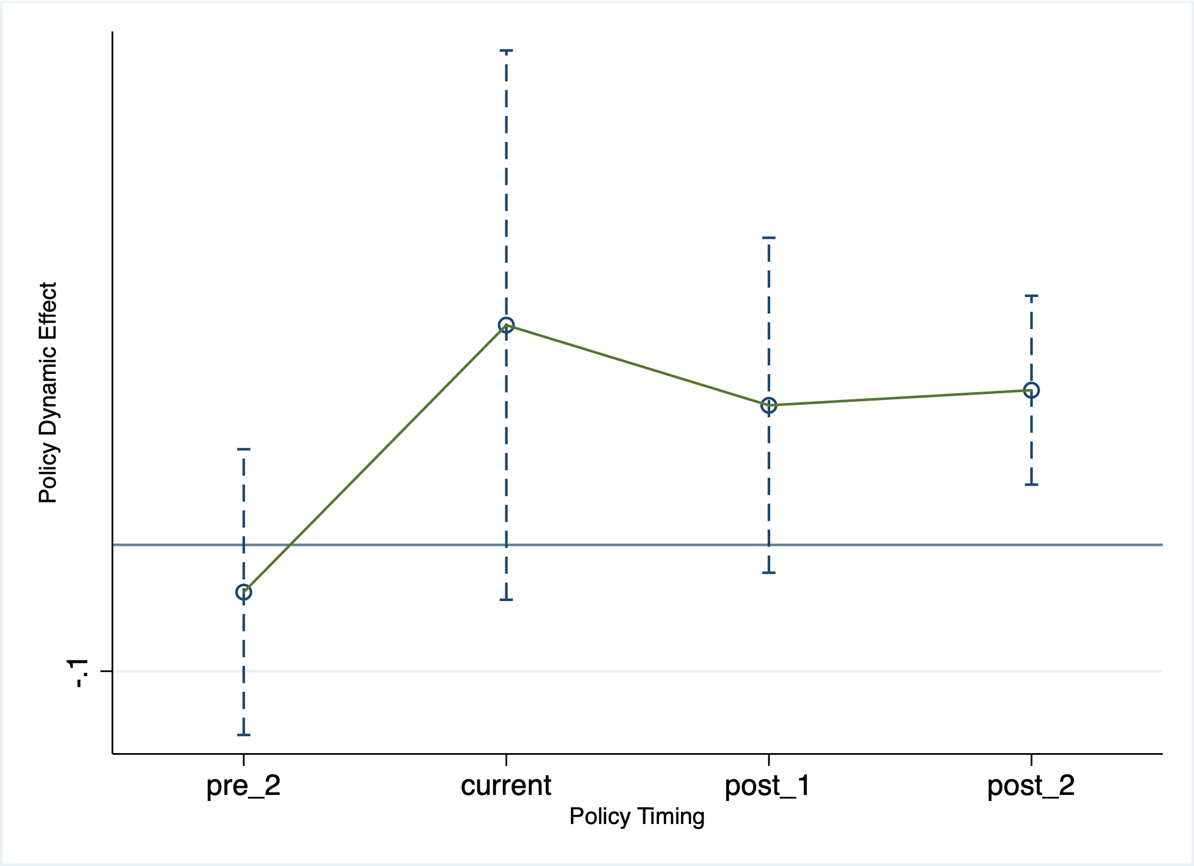


Fig. B.2 Results of $QTY\_Watch$ %

Appendix C: Placebo Test

Fig. C.1 Results of quantity of Access antibiotics %

Note: The X-axis represents 500 randomly generated estimated coefficients. Hollow circles represent the P-values of the estimated coefficients, and the solid line represents the kernel density distribution of the estimated coefficients. The vertical line on the left side represents the estimated coefficient for the actual policy.

Fig. C.2 Results of quantity of Watch antibiotics %

Appendix D:

Table. S1. Impact of hospital payment reform on hospital antibiotic utilization under China's classification system using multivariate difference-in-differences analysis

|  |  | Model 1^a^ |  |  |  |  | Model 2^b^ | |  | |  | |
| --- | --- | --- | --- | --- | --- | --- | --- | --- | --- | --- | --- | --- |
|  | $\beta$ | 95% | CI | *P* |  | $\beta$ | 95% | CI | | *P* | |  |
| Non-restricted quantity % | -0.03 | -0.11 | 0.04 | 0.355 |  | -0.03 | -0.11 | 0.04 | | 0.355 | |  |
| Non-restricted expenditure % | -0.04 | -0.10 | 0.03 | 0.291 |  | -0.05 | -0.12 | 0.02 | | 0.142 | |  |
| Restricted quantity% | 0.03 | -0.04 | 0.09 | 0.423 |  | 0.04 | -0.02 | 0.11 | | 0.207 | |  |
| Restricted expenditure % | 0.01 | -0.06 | 0.07 | 0.863 |  | 0.01 | -0.06 | 0.09 | | 0.710 | |  |
| Non-restricted to Restricted quantity ratio | -6.27 | -13.44 | 0.89 | 0.088 |  | -8.70 | -16.39 | -1.02 | | 0.028 | |  |
| Non-restricted to Restricted expenditure ratio | 25.43 | -42.32 | 93.18 | 0.463 |  | 24.80 | -48.97 | 98.58 | | 0.511 | |  |

Abbreviations: CI, confidence interval.

^a^In model 1, control variable is hospital grade.

^b^In model 2, control variables included hospital grade, number of hospital beds per thousand people, number of doctors per thousand people, Health expenditure per capita, and proportion of government financial support.

^c^Since their distributions were skewed, the total quantity and total expenditure were ln transformed to be included in the regression model.
